# A model-agnostic approach for understanding heart failure risk factors

**DOI:** 10.1186/s13104-021-05596-7

**Published:** 2021-05-17

**Authors:** Seyed M. Miran, Stuart J. Nelson, Qing Zeng-Treitler

**Affiliations:** Biomedical Informatics Center, School of Medicine and Health Sciences, George Washington University, Washington, DC USA

**Keywords:** Explainable AI, Model-agnostic approach, Heart failure

## Abstract

**Objective:**

Understanding the risk factors for developing heart failure among patients with type 2 diabetes can contribute to preventing deterioration of quality of life for those persons. Electronic health records (EHR) provide an opportunity to use sophisticated machine learning models to understand and compare the effect of different risk factors for developing HF. As the complexity of the model increases, however, the transparency of the model often decreases. To interpret the results, we aimed to develop a model-agnostic approach to shed light on complex models and interpret the effect of features on developing heart failure. Using the HealthFacts EHR database of the Cerner EHR, we extracted the records of 723 patients with at least 6 yeas of follow up of type 2 diabetes, of whom 134 developed heart failure. Using age and comorbidities as features and heart failure as the outcome, we trained logistic regression, random forest, XGBoost, neural network, and then applied our proposed approach to rank the effect of each factor on developing heart failure.

**Results:**

Compared to the “importance score” built-in function of XGBoost, our proposed approach was more accurate in ranking the effect of the different risk factors on developing heart failure.

**Supplementary Information:**

The online version contains supplementary material available at 10.1186/s13104-021-05596-7.

## Introduction

Heart Failure (HF) is a serious problem for public health and the economy in the United States[1–3]. Coronary artery disease and hypertension significantly increase the risk of developing HF [4, 5]. Results from the Framingham Heart Study showed that the risk of developing HF among patients with hypertension is up to three times more than that of normotensive people [6]. atrial fibrillation [7], chronic obstructive pulmonary disease (COPD)[8, 9], chronic kidney disease (CKD) [10], anemia [12], asthma [11, 13], arthritis [14], depression [15], and cancer [16] have also been identified to correlate with HF.

Many research studies have compared the effect of different comorbidities on developing HF [17]. The results of these studies, however, are not always consistent. Coronary heart disease, diabetes, and hypertension have been identified as the most important risk factors [18]. Levy and his colleagues [6] monitored 5,143 people for 20.1 years and reported that hypertension was the biggest risk factor for developing HF. In a separate study, the authors found that coronary heart disease had the biggest effect on developing HF [19]. Diabetes and HF, which are often called “twin epidemics”, are highly correlated [20]. Comparing the effect of the above-mentioned comorbidities on the risk of developing HF among patients with type 2 diabetes is thus of particular interest for prevention and treatment purposes.

Electronic Health Records (EHR) are increasingly employed by clinical researchers. Using EHR data, machine learning algorithms can help to understand the effect of different comorbidities on the clinical outcome [21, 22]. In the current study, we used HealthFacts, an EHR-based database developed and maintained by Cerner Corporation, to understand and interpret the effect of age and different well-known comorbidities on developing HF among patients with type 2 diabetes.

Different modeling methods, such as logistic regression, or machine learning methods, such as random forest, XGBoost, and neural networks, can be used for our binary classification task of developing HF. Given the increasing amount of EHR data, more sophisticated models may improve the assessment of the risk of a clinical outcome. However, the most powerful predictive models, such as the ensemble model and deep neural network, are not interpretable [23]. Identifying the most significant factors for the risk of HF among patients with diabetes requires improvement of the global interpretability of machine learning algorithms.

Different researchers from different fields of study have sought to understand the effect of individual features on an outcome when the underlying relationship is nonlinear and nonmonotonic [24, 25]. One approach is to perturb the values of a given feature with random numbers while keeping the other features fixed in order to investigate the relationship between the change of the response and to calculate the “situational importance” of each feature [26, 27]. The main difficulty with this approach are that many randomly generated values do not happen in the real world, and that the approach does not consider the underlying dependent relationship between variables.

The Shapley value, based on a study by Shapley decades ago [28], has been used to investigate the global effect of each individual feature on the response. For a feature, its Shapley value is the average marginal contribution of that feature across all possible combinations of all features. Although the Shapley value can help rank the importance of the features, it is computationally very expensive especially when the number of features is large. Like most of the permutation-based algorithms, its other drawback is that it may include unrealistic instances in the computation in cases where the features are correlated.

Another model-agnostic approach to compare and understand the global effect of features on the response is through the use of a “global surrogate.” The idea behind this is to train an interpretable model (such as a linear model) on the features and their predictions from the black-box model to approximate the underlying model. Local interpretable model-agnostic explanations (LIME) is a method that is capable of providing both local and global interpretability using this approach [29].

Although all the methods mentioned above can shed light on a black-box model, all of them have some degree of randomness and do not necessarily lead to the same result when repeated for the same underlying model. The LIME and Shapley value methods are very computationally expensive. Developing an approach that is deterministic in results with less computational burden can make a significant contribution to the field.

## Main text

### Methods

#### Data procurement and preparation

In this research study, we used the Health Facts database, which has more than 400 million encounters from 689 different hospitals. We first identified patients with type 2 diabetes using ICD-9 and ICD-10 codes, while requiring at least one encounter without the diabetes ICD codes before the first diabetes visit. The date of the first encounter with the ICD diabetes codes was considered as the index date. We excluded patients who had developed HF prior to the index date. We required that each patient have at least six years of follow up with at least one encounters each year. Most patients in the database do not have long term follow-up, thus we could identify only 723 patients. The average age for patients who developed HF was 68 years old (min = 40, max = 90) and the average age for patients who did not develop HF was 64.37 years old (min = 40, max = 90). The cohort characteristics at the end of the follow-up time can be seen in Additional file 1: Table S1.

#### Model building

Since the data size was relatively small, we bootstrapped it with replacement and increased the size to 10,723. We randomly selected 80% of the encounters for training and 20% for testing purpose. Using the Sklearn Python package, we fitted logistic regression, random forest, XGBoost models, and used the Keras python package to fit a deep neural network model to the data. We tuned hyperparameters through five-fold cross-validation. For the neural nets, the best model by the ROC-AUC measurement, was a fully connected network with four hidden layers and different nodes, ranging from 5 to 22 nodes, implementing sigmoid and ReLu activation functions. To prevent overfitting, dropout at a rate of 0.25 was applied to each hidden layer. Finally, using the test data, we found that ROC-AUC for XGBoost was greater than the other three methods. The only transparent method, logistic regression, was outperformed by the black-box methods. The ROC-AUC of the different models are as follows: Logistic regression: 0.62; Neural network: 0.68; Random forest: 0.74; XGBoost: 0.91. In order to understand the effect of different features on the risk of HF, we need to interpret the black-box models.

#### Effect score

We developed a model-agnostic metric called “effect score” to compare and interpret the effect of each feature on the risk of the clinical outcome. This method calculates how the logit of the output changes if the current value of feature ${\varvec{i}}$ $({{\varvec{x}}}_{{\varvec{i}}}^{{\varvec{c}}})$ changes from a chosen reference value $\left({{\varvec{x}}}_{{\varvec{i}}}^{{\varvec{r}}}\right)$ for that feature, e.g., a normal value for a lab test. In order to lower the chance of incorporating unrealistic possibilities by using random values, the value of feature ${\varvec{i}}$ is replaced only with other observed values for the same feature. Given that there are ${\varvec{m}}$ features and ${\varvec{n}}$ observations in the system, the algorithm for computing the effect score for feature ${{\varvec{x}}}_{{\varvec{i}}}$ can be seen in Table 1.

**Table 1:** Different steps to compute the effect score

Step number	Steps
1	fit a machine learning model (for a neural network model, the activation function of the output layer needs to be a sigmoid function)
2	determine a reference value ${x}_{i}^{r}$
3	$\left.{es}_{i,j}=logit\left(f\left({x}_{1}^{j},\dots ,{x}_{i}^{j},\dots ,{x}_{n}^{j}\right)\right)-logit(f({x}_{1}^{j},\dots ,{x}_{i}^{r},\dots ,{x}_{n}^{j})\right)$ - where $f(.)$ is the prediction of the probability of the positive class by the model - if ${x}_{i,j}={x}_{i,k}$, then consider the average of them
4	${ES}_{i}= \sum_{j=1}^{n}|{es}_{i,j}|$
5	For a continuous feature, plot ${es}_{i,j}$ against the value of ${x}_{i, j \epsilon n \left\{obs\right\}}$ to depict the effect of ${x}_{i}$ at different values on the output with respect to the reference
6	Rank ${ES}_{i}$ of categorial and continuous features to compare strength of different features

### Results and discussion

Since the XGBoost model resulted in the highest area under the ROC curve, we used that model for understanding HF risk factors. As there was only one continuous variable, “age”, we only used the “effect score” algorithm for comparing the effects of categorical variables on the risk of HF. The result of the algorithm can be seen in Table 2.

**Table 2:** Effect scores for categorical features

Feature	Effect score
Ischemic heart disease (IHD)	1.75
Hypertension (HTN)	1.68
Atrial fibrillation (AF)	1.52
Chronic obstructive pulmonary disease (COPD)	1.52
Cancer	1.48
Chronic kidney disease (CKD)	1.40
Anemia	1.36
Asthma	1.34
Arthritis	1.02
Depression	1.00

As it can be seen in Table 2, the highest score = 1.75 is achieved with ischemic heart disease, thus it is considered the most influential individual risk factor for developing HF. This score can be interpreted as meaning that if all other features for $patient i$ and $patient j$ are the same and $patient i$ has ischemic heart disease but $patient j$ has not, the logit of developing HF for $patient i$ is on average 1.75 more than that of $patient j$. The other scores can be interpreted in a similar fashion.

One might argue that the “effect score” is more helpful if the underlying model is a neural network model. Ensemble methods, including XGBoost, can provide an “importance score” that ranks the degree of influence of different features on the output. Difficulty with the “importance score” occurs when the predictors are correlated, like those in this case; a result that leads noninfluential predictors to be preferred to significant ones [30]. When we applied the importance metric through Sklearn library in Python, this issue was observed in our use case as well. The ranking of the features from that method is as follows: CKD, COPD, anemia, IHD, Depression, AF, HTN, cancer, and Arthritis. Using this metric, hypertension, long known as a very important factor in the development of HF [6, 18, 19] has less importance than depression. It is highly doubtful that depression is clinically a more significant risk factor than hypertension. This ranking is not consistent with the current clinical.

knowledge. Additionally, it should be noted that the Sklearn approach provides no avenue for interpretation of the meaning of the scores.

**Fig. 1:**
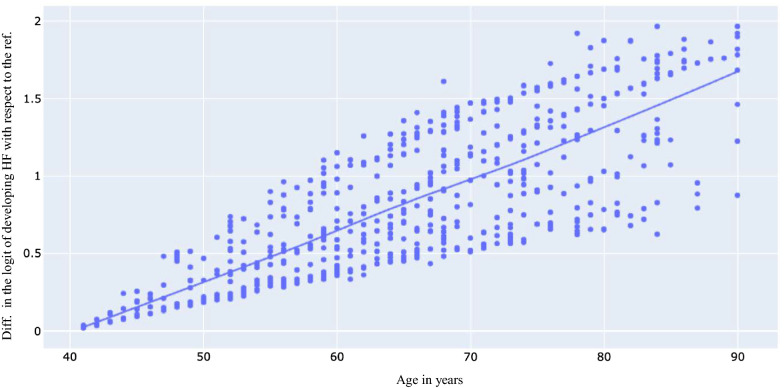
Visualization of relationship between age and heart failure

Another use of our proposed approach is to understand the nonlinear relationship between a continous feature and the output. In Fig. 1, it can be seen that, as it was expected [31], the risk of HF increases as the age increases. Each point denotes the difference between the logit of risk of HF for $patient i$ with that of $patient j$, who has the same comorbidities but is 40 years old. The LOWESS (locally weighted scatterplot smoothing) technique was used to show the trend between the age and risk of HF on the population level. The figure suggests the relationship between the age and the risk of HF is linear. The diversion of observations at the same age indicates that there is a correlation between the age and different comorbid conditions.

### Summary and conclusion

Enhancing interpretation of machine learning models can contribute to a better understanding of clinical events. In this paper, we propose a model-agnostic approach to explore how a complex machine learning model can be used to investigate risk factors associated with developing HF among patients with type 2 diabetes. Our approach enables a researcher to interpret the global effect of each individual feature on the outcome; compare the significance of different individual categorical variables; appreciate a nonlinear nonmonotonic relationship between a continuous feature and outcome; visualize the effect of each individual observation on the outcome; and fit a trendline through locally weighted scatterplot smoothing to understand the global effect of that feature.

## Limitations

• In future work, the proposed algorithm should be implemented using a higher quality EHR database with larger patient samples.
• This algorithm should not be used to compare the effect of a continuous variable on the outcome with that of a categorical variable.
• When the number of levels of two categorical variables are far different, the results from this algorithm may be biased.

## Supplementary Information

**Additional file1**: **Table S1** Patient characteristics.

## Data Availability

The datasets used as input are the property of Cerner corporation, from whom they are available.
