# Supplementary material for: A model-agnostic approach for understanding heart failure risk factors
Source: BMC Res Notes. 2021 May 17;14:184. doi: 10.1186/s13104-021-05596-7 (PMC8130447; doi:10.1186/s13104-021-05596-7)
Supplement: Supplementary file 1 — Additional file1: Table S1 Patient characteristics. [file 13104_2021_5596_MOESM1_ESM.docx]

| TABLE S1. PATIENT CHARACTERISTICS | |
| --- | --- |
| **Data point** | **Num. of cases** |
| Total Patients | 723 |
| Hypertension (HTN) | 622 (86.03%) |
| Ischemic Heart Disease (IHD) | 358 (49.52%) |
| Atrial Fibrillation (AF) | 116 (16.04%) |
| Chronic Kidney Disease (CKD) | 217 (30.01%) |
| Anemia | 102 (14.11%) |
| Arthritis | 496 (4.63%) |
| Asthma | 120 (16.6%) |
| Chronic Obstructive Pulmonary Disease (COPD) | 158 (21.85%) |
| Cancer | 103 (14.25%) |
| Depression | 179 (24.76%) |
| Heart Failure (HF) | 134 (18.53%) |
